# A deep learning approach for successful big-bubble formation prediction in deep anterior lamellar keratoplasty

**DOI:** 10.1038/s41598-021-98157-8

**Published:** 2021-09-17

**Authors:** Takahiko Hayashi, Hiroki Masumoto, Hitoshi Tabuchi, Naofumi Ishitobi, Mao Tanabe, Michael Grün, Björn Bachmann, Claus Cursiefen, Sebastian Siebelmann

**Affiliations:** 1 Division of Ophthalmology, Department of Visual Sciences, Nihon University School of Medicine, Ohyaguchikami-machi 30-1, Itabashi-ku, Tokyo, 173-8610 Japan; 2 Department of Technology and Design Thinking for Medicine (DT2M), Hiroshima University, Hiroshima, Japan; 3 Department of Ophthalmology, Jichi Medical University, Shimotsuke, Tochigi Japan; 4 Xeno-Hoc, Shinjyuku, Tokyo Japan; 5 Department of Ophthalmology, Tsukazaki Hospital, Himeji, Japan; 6 Department of Ophthalmology, University of Cologne, Cologne, Germany; 7 MVZ ADTC Mönchengladbach/Erkelenz, Erkelenz, Germany

**Keywords:** Medical research, Optics and photonics

## Abstract

The efficacy of deep learning in predicting successful big-bubble (SBB) formation during deep anterior lamellar keratoplasty (DALK) was evaluated. Medical records of patients undergoing DALK at the University of Cologne, Germany between March 2013 and July 2019 were retrospectively analyzed. Patients were divided into two groups: (1) SBB or (2) failed big-bubble (FBB). Preoperative images of anterior segment optical coherence tomography and corneal biometric values (corneal thickness, corneal curvature, and densitometry) were evaluated. A deep neural network model, Visual Geometry Group-16, was selected to test the validation data, evaluate the model, create a heat map image, and calculate the area under the curve (AUC). This pilot study included 46 patients overall (11 women, 35 men). SBBs were more common in keratoconus eyes (KC eyes) than in corneal opacifications of other etiologies (non KC eyes) (*p* = 0.006). The AUC was 0.746 (95% confidence interval [CI] 0.603–0.889). The determination success rate was 78.3% (18/23 eyes) (95% CI 56.3–92.5%) for SBB and 69.6% (16/23 eyes) (95% CI 47.1–86.8%) for FBB. This automated system demonstrates the potential of SBB prediction in DALK. Although KC eyes had a higher SBB rate, no other specific findings were found in the corneal biometric data.

## Introduction

Deep anterior lamellar keratoplasty (DALK) has been a successful treatment option for corneal stromal diseases such as keratoconus or corneal opacification^1,2^. In comparison to the former gold standard, penetrating keratoplasty (PK), DALK has certain advantages in terms of time to visual recovery, endothelial cell density loss, and low immunological graft rejection rates^3^.

However, DALK involves a steep learning curve, especially when exposing the Descemet’s membrane (DM). Although different techniques such as air injection or viscoelastic devices to expose the DM have been introduced, these techniques are not always reproducible and sometimes lead to separation failure between the corneal stroma and the posterior corneal complex^4–7^. Even experienced surgeons could face a risk of failure, which could result in incomplete DM exposure or rupture. The big-bubble technique, first introduced by Dr. Anwar (Anwar’s big-bubble technique), is the most popular method for exposing Descemet membranes^8^. However, despite employing this technique, a failure rate of approximately 10% for intraoperative conversion from DALK to PK due to DM rupture remains^6^. New surgical approaches, such as the microbubble incision technique and a pentacam-based approach, have reduced the conversion rate^9,10^. Nonetheless, predicting the probability of successful big-bubble formation (SBB) before the beginning of the procedure could be extremely helpful. The success of the big-bubble technique depends on the morphology of the cornea and the extent of the pathological structure, such as the depth or extent of a scar in the corneal stroma^11^. Structural changes in the corneal tissue can be non-invasively recorded with about histological resolution using optical coherence tomography (OCT). The images thus generated are excellently suited for further image data analysis and can be collected preoperatively in almost all patients before DALK.

In recent times, machine learning analysis of clinical images, such as from OCT scans, has gained significant attention in ophthalmology and various other medical fields^12,13^. Although several previously published studies have already described “predictive factors” for successful big-bubble formation, it has not been reported using an automatic judgement system^14–16^. Therefore, this study aimed to evaluate the predictability of successful big-bubble formation in DALK using machine learning and to compare the results to preoperatively assessed biometric values.

## Results

### Patient characteristics

Table 1 summarizes the patients’ characteristics. In this study, 46 eyes (24 left eyes) from 46 patients were included (11 women and 35 men; mean age 48.1 ± 17.7 years [mean ± SD]). The underlying disease was keratoconus (KC eyes) in 29 patients and corneal opacification due to other etiologies (non-KC eyes) in 17 eyes. Eyes that had been converted to PK due to intraoperative DM perforation were excluded from this study.

**Table 1:** Patient characteristics.

	Total
Number of eyes	46
Sex: female (%)/male (%)	11 (23.9%)/35 76.1(78.2%)
Age (years) (mean ± SD)	48.1 ± 17.7
**Etiology**	
Keratoconus	29
Corneal opacity	17
**Grouping**	
SBB (successful big bubble)	23
FBB (failed big bubble)	23

### Analysis of clinical data

A total of 46 eyes were analyzed. Regarding the underlying disease, SBBs were more common in KC eyes than in non-KC eyes (*p* = 0.006). Although K values (Kmax or Kmean) tended to be higher in the SBB group than in the failed big-bubble (FBB) group, this difference was not statistically significant (*p* = 0.097 [Kmax], *p* = 0.116 [Kmean], respectively). Similarly, corneal thickness values (central corneal thickness and thinnest corneal thickness) were smaller in the SBB group but did not differ significantly (p = 0.932 and p = 0.783, respectively). The FBB group tended to have higher densitometry than the SBB group, but this difference was not significant (p = 0.119). A summary of biometric data is shown in Table 2.

**Table 2:** Comparison of preoperative biometric values between the SBB and NBB groups.

	All eyes (n = 55)	SBB group (n = 28)	FBB group (n = 27)	p value (FBB vs. NBB)
Etiology; proportion of KC	29/46	19/23	10/23	0.006^†^
K max (D) (mean ± SD)	67.6 ± 15.0	71.3 ± 14.3	63.9 ± 14.8	0.097
K mean (D) (mean ± SD)	56.1 ± 11.0	58.7 ± 10.9	53.6 ± 10.4	0.116
Central corneal thickness (µm) (mean ± SD)	484 ± 164	482 ± 179	487 ± 148	0.932
Thinnest corneal thickness (µm) (mean ± SD)	416 ± 138	410 ± 126	421 ± 150	0.783
Densitometry	68.1 ± 26.4	61.8 ± 24.6	74.2 ± 26.7	0.119

SBB, successful big bubble; FBB, failed big bubble; SD, standard deviation.

^†^Pearson’s chi-square test.

### ROC curve for eye-by-eye determination

**Figure 1:**
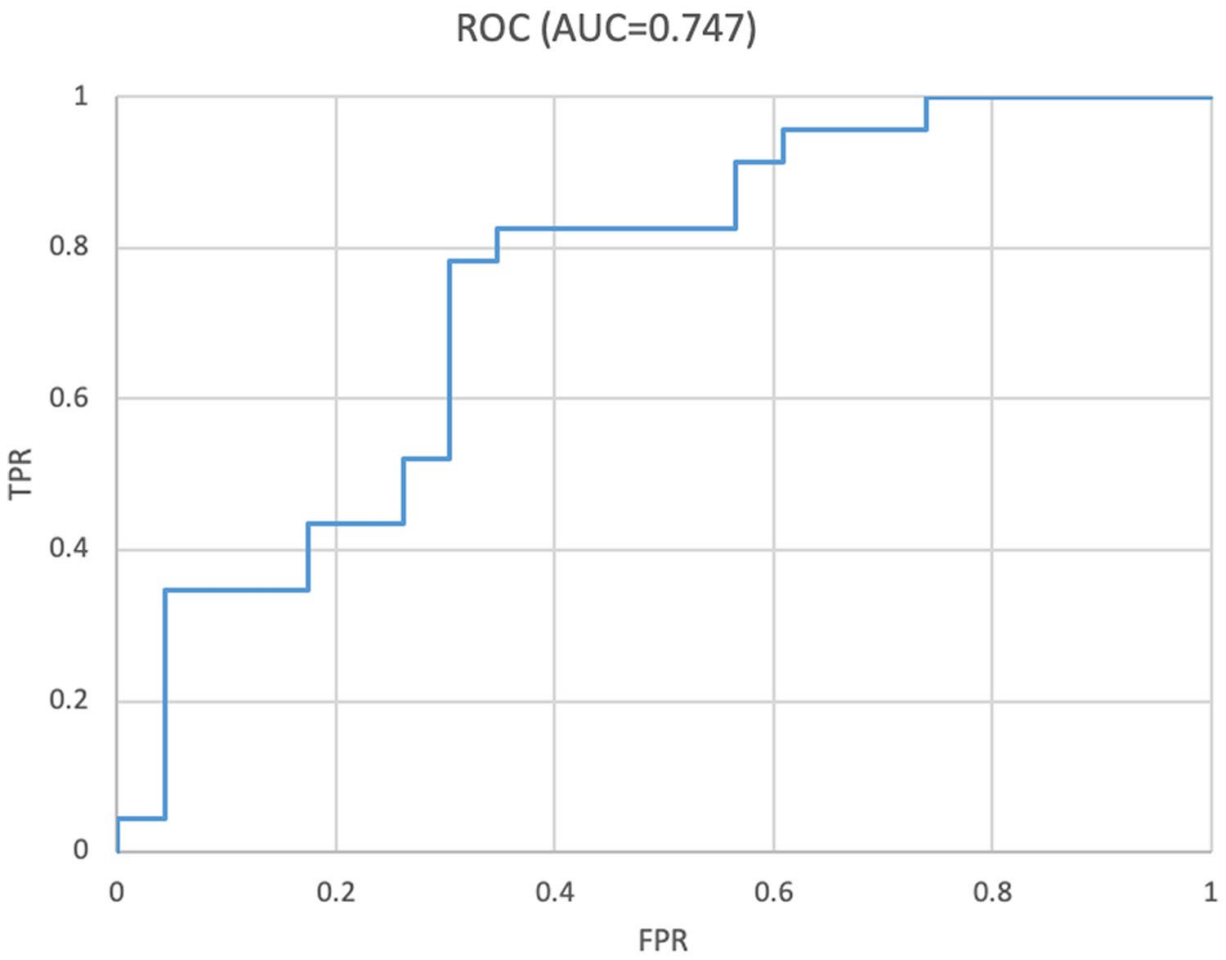
Receiver operating characteristic (ROC) curve for determination. The AUC was 0.746 (95% confidence interval [CI] 0.603–0.889).

The AUC was 0.746 (95% confidence intervals [CI] 0.603–0.889) (Fig. 1). The determination success rate was 78.3% (18/23 eyes) (95% CI 56.3–92.5%) for SBB and 69.6% (16/23 eyes) (95% CI 47.1–86.8%) for FBB.

## Discussion

Automated image recognition using deep learning methods is becoming increasingly important in the decision making, planning, and execution of ophthalmic surgeries. While there are numerous studies on the retina, there are very few studies on the anterior eye segments, especially the cornea^17,18^. Nevertheless, it has been shown that artificial intelligence and image data obtained by OCT can be used to detect and classify certain corneal diseases, and even predict the probability of the need for future keratoplasty^19^. Similar findings have been described before cataract surgery or for the detection of KC^20,21^. The present study confirms that, even in complex situations such as predicting the success of a particular surgical maneuver (BB formation in DALK), intelligent algorithms can make very good predictions. KC eyes had a better success rate of BB formation than those with corneal opacification, although a selection of pre-selected, established parameters did not reveal a statistically significant difference between the SBB and FBB groups.

In this study, we aimed only to test if we could develop a relatively precise algorithm to predict the formation of a big bubble with few data, without using already known associating factors. Given that the results indicate the determination success rate was 78.3% for SBB and 69.6% for FBB, we demonstrated that it was possible to develop the automatic judgement algorithm. However, the accuracy could be increased using a larger sample size with the same indications. Thus, the limitations of this pilot study are that, due to the small sample size, the accuracy of individual and special indications for DALK could not be elucidated, such as herpetic scars, traumatic scars, exclusively KC, or corneal dystrophies.

Therefore, in the future, it would be useful to collect OCT data from several centers to develop synergies and perform subgroup analyses by using the developed algorithm here. This could be facilitated by cross-country and cross-device web platforms. Alternatively, large amounts of available data would overcome this limitation. In the present study, the success rate of one type of BB (Anwar’s big-bubble) by only two surgeons was evaluated. Therefore, these data are probably not easily transferable to other surgeons or techniques; nevertheless, this pilot study shows the possibilities of such analysis using artificial intelligence. However, one of the main points of criticism for the clinical implementation could be that the criteria of the algorithm used to make its relatively precise statements are not sufficiently clear. In the future, similar algorithms combined with modern intraoperative imaging technologies, such as microscope-integrated optical coherence tomography and real-time intraoperative pattern recognition algorithms could help ophthalmic surgeons not only before or after surgery, but also during the execution of surgical maneuvers^22^.

In conclusion, this pilot study demonstrated the possibility of predicting of SBBs. Future studies should aim to integrate larger data sets using various imaging modalities.

## Materials and methods

### Study design

In this retrospective study, the OCT images of all patients who underwent DALK between March 2013 and July 2019 were analyzed. Images were acquired using a Spectral Domain OCT device (SD-OCT, Heidelberg Engineering, Heidelberg, Germany). The study complied with the ethical standards of the Declaration of Helsinki and was approved by the institutional review board of the University of Cologne, Cologne, Germany (File Number 15–301). Informed written consent was obtained from all participants before enrollment.

### Surgical technique

All surgeries were performed under general anesthesia by two experienced surgeons^23^. DALK was performed in a standardized fashion using Anwar’s big-bubble technique as previously described^8^. Briefly, the host cornea was marked using a trephine (Katena, Denville, USA) at 7.5–8.0 mm, the DM exposed, and the host stroma carefully removed. The donor graft without DM was prepared using a donor punch (Katena, Denville, USA) at 7.75–8.5 mm and then secured with a 10–0 nylon suture by either a running suture or interrupted suture technique. In cases where no big bubbles could be created, the DM was exposed by dissecting the stroma in layers using a manual dissection technique^5^.

### Grouping by intraoperative procedures

Patients who underwent DALK were divided into two groups: (1) successful big-bubble group or (2) failed big-bubble group.

### Collection of preoperative biometric data

To obtain preoperative biometrical data, the eyes were examined preoperatively using the Scheimpflug tomography system (Pentacam HR, Oculus GmbH, Wetzlar, Germany), preoperative corneal thickness values (central corneal thickness, thinnest corneal thickness), keratometric values (Kmax, Kmin), and the corneal densitometry was thereon compared between the two groups (SBB and FBB).

### Dataset and grouping for deep learning

For constructing the artificial intelligence-based image analysis system, the following two categories were defined: SBB vs. FBB. The preoperative corneal cross-sectional OCT images captured from SD-OCT volume scans or manual single scans were analyzed.

In this study, the K-fold cross-validation (K = 5) method was applied^24,25^. Image data were divided into K groups. The K-1 group was used as training data and the remaining group as evaluation data. The process was repeated K times until all groups were used as evaluation data. During training, the following data expansion was performed for each epoch: rotation, displacement, shear, enlargement, vertical inversion, horizontal inversion, two types of brightness adjustment, two types of gamma correction, blurring, histogram flattening, and two types of noise load. Training was performed for a deep convolutional neural network.

### Model construction

For the original image, the image consisting of 1024 × 242 pixels was read as the RGB channel. First, the image was resized to 224 × 224 pixels. The pixel value consisting of 8 bits (range of 0–255) was divided by 255 and normalized to the range of 0–1.

In this study, we tested various deep neural network (DNN) models of DenseNet121, EfficientNetB0, and Visual geometry group-16 (VGG-16) and finally selected VGG-16 because of its balanced values (data not shown). This type of DNN is known to automatically learn the local features of an image to generate a classification model^26–30^. VGG-16 consists of five blocks and three fully connected layers. Each block consists of several convolutional layers that automatically extract features and max-pooling layers that reduce position sensitivity and improve generalization performance^31^.

The convolution layer stride was equal to one and the layer padding was set to be the same. As a result, the convolutional layer only grasped the image features and did not downsize. The activation function was set as rectified linear unit (ReLU) to avoid the problem of gradient disappearance^32^. The stride of the max-pooling layer was set to two. After Block 5, there were flatten layers and two fully connected layers. Spatial information was removed from the feature vector extracted by the flatten layer. The fully connected layer compressed the information. Finally, the probability of each class was evaluated by passing a function called softmax, and target images classified. Fine-tuning (or full retraining) was performed to increase the learning speed so that high performance could be achieved with a small amount of data^33^. The parameters of blocks 1–4 used ImageNet as initial values.

The parameters of each layer were updated using the momentum stochastic gradient descent algorithm (updated using learning rate = 0.0005, initial term = 0.9)^34,35^. In addition, training and verification were performed using Keras, a Python TensorFlow Application Programming Interface (API) (https://www.tensorflow.org/).

### Deep learning

Using the Score-CAM method^36^, we created a heat map image showing where the DNN was concentrated. The target layer was set as the maximum pooling layer of block 5, and the ReLU function was used to correct the loss function during backpropagation.

The performance evaluation indices were the area under the curve (AUC) and the correct answer rate in the successful and unsuccessful eye. The calculation method was as follows. First, since the probability of successful DM separation can be discerned by the neural network output, in each image, an image output with a value > 0.5 was regarded as successful, whereas images with a low value were unsuccessful. When the ratio of the number of successfully determined images in each eye exceeded a certain threshold, it was determined that the eye will succeed, and the AUC was calculated by adjusting the threshold.

The ROC curve used to determine the AUC was created by defining the point at which the value used to indicate SBB positively exceeded the threshold cutoff value output from the softmax function. We created 100 ROC curves from 100 patterns with 10% thinned out; thus, this model was applied to only 90% of the test data. A total of 100 AUCs were calculated from each ROC curve, and a 95% CI was obtained by assuming a normal distribution and average standard deviation. In addition, we set the threshold value that maximized the performance using the Youden index and then calculated the correct answer rate in the successful and unsuccessful eyes^37^. The CIs of sensitivity and specificity were calculated assuming a binomial distribution.

### Statistical analysis

Statistical analyses were performed using JMP Pro software version 14.0.0 (SAS Institute, Cary, NC, USA). To compare the continuous variables in each group, we used either one-way analysis of variance (ANOVA) or Mann–Whitney U test, while nominal variables such as patient sex and operated eye were compared using Pearson’s chi-square test.

## Data Availability

The data that support the findings of this study are available from the corresponding author [T.H], upon reasonable request.
